# Supplementary material for: Seminoma and Embryonal Carcinoma Footprints Identified by Analysis of Integrated Genome-Wide Epigenetic and Expression Profiles of Germ Cell Cancer Cell Lines
Source: PLoS One. 2014 Jun 2;9(6):e98330. doi: 10.1371/journal.pone.0098330 (PMC4041891; doi:10.1371/journal.pone.0098330)
Supplement: File S1 — ZIP file containing DMRforPairs output for significant regions. Please start from the html files. (ZIP) [file pone.0098330.s008.zip › figures/19867.pdf]

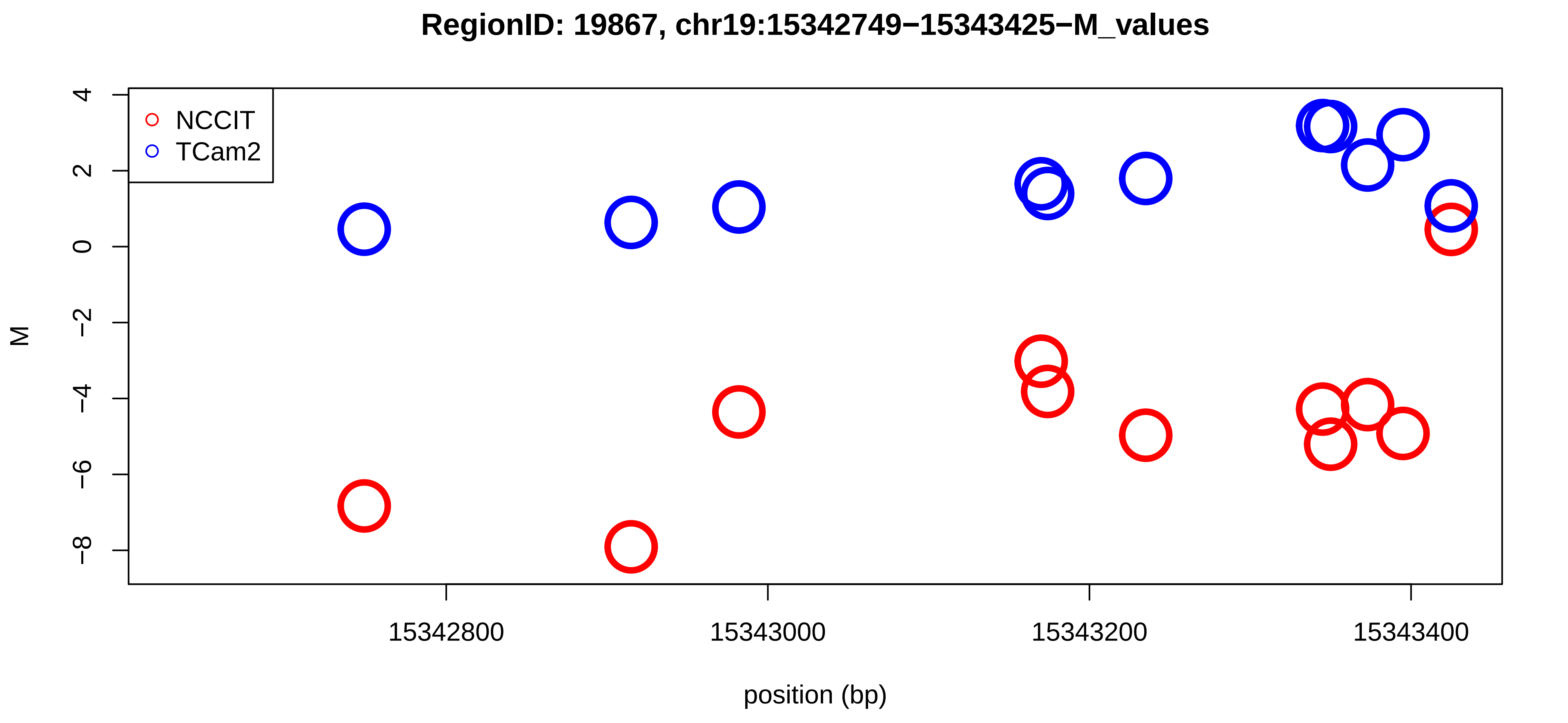
RegionID: 19867, chr19:15342749−15343425−M_values
NCCIT
TCam2
M
4
2
0
−2
−4
−6
−8
15342800
15343000
15343200
15343400
position (bp)

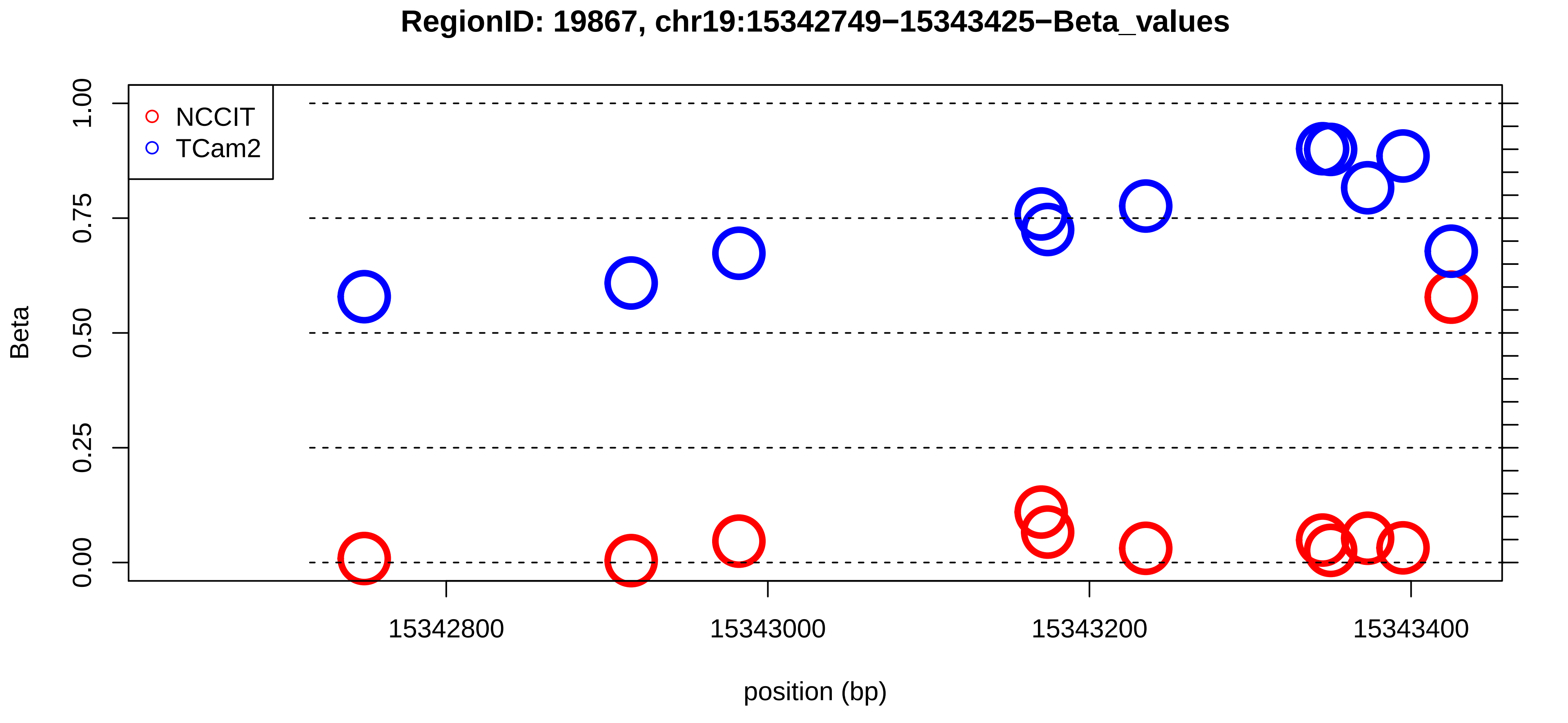
RegionID: 19867, chr19:15342749−15343425−Beta_values
NCCIT
TCam2
Beta
1.00
0.75
0.50
0.25
0.00
15342800
15343000
15343200
15343400
position (bp)

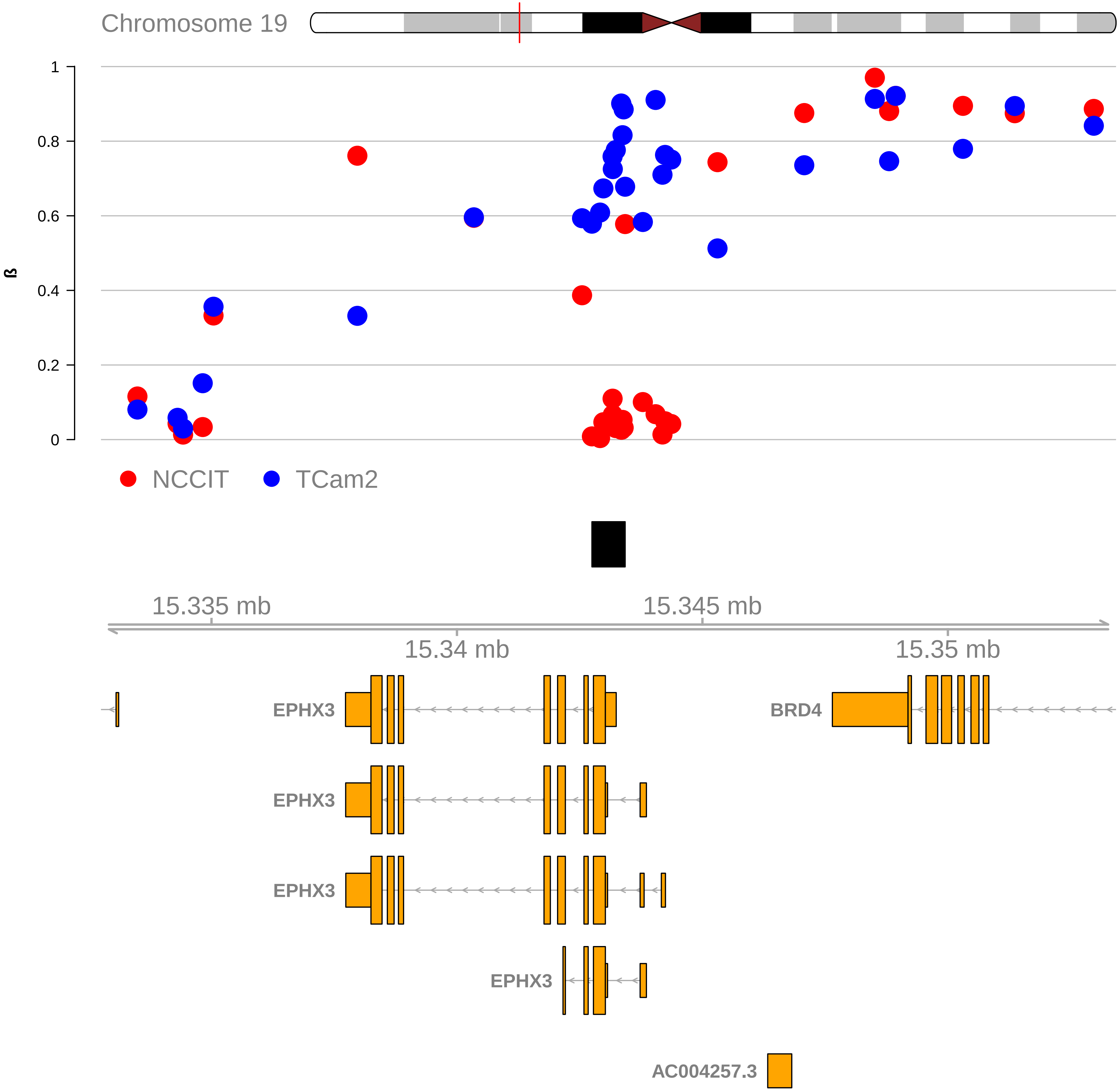

Chromosome 19
1
0.8
0.6
0.4
0.2
0
ß
NCCIT
TCam2
15.335 mb
15.345 mb
15.34 mb
15.35 mb
EPHX3
BRD4
EPHX3
EPHX3
EPHX3
AC004257.3
